# Aromatic Volatile Compounds of Essential Oils: Distribution, Chemical Perspective, Biological Activity, and Clinical Applications

**DOI:** 10.1002/fsn3.70825

**Published:** 2025-08-25

**Authors:** Hamdoon A. Mohammed, Ghassan M. Sulaiman, Ali Z. Al‐Saffar, Mayyadah H. Mohsin, Riaz A. Khan, Noora A. Hadi, Shahad Basil Ismael, Fatma Elshibani, Ahmed Ismail, Mosleh M. Abomughaid

**Affiliations:** ^1^ Department of Medicinal Chemistry and Pharmacognosy College of Pharmacy, Qassim University Buraydah Saudi Arabia; ^2^ College of Applied Sciences University of Technology Baghdad Iraq; ^3^ Department of Molecular and Medical Biotechnology College of Biotechnology, Al‐Nahrain University Baghdad Iraq; ^4^ Department of Plant Biotechnology, College of Biotechnology Al‐Nahrain University Baghdad Iraq; ^5^ Department of Pharmacognosy Faculty of Pharmacy, University of Benghazi Benghazi Libya; ^6^ Pharmacognosy Department Faculty of Pharmacy, Fayoum University Fayoum Egypt; ^7^ Department of Medical Laboratory Sciences College of Applied Medical Sciences, University of Bisha Bisha Saudi Arabia

**Keywords:** aromatic volatile compounds of essential oils (ACEO), biogenesis, biological activity, chemistry, clinical applications, essential oil

## Abstract

Volatile, or essential, oils are the principal odorous components of certain plant species and constitute aliphatic and aromatic compounds in the oils' natural mix. The aromatic compounds are present in significant proportions in the oils of several plant species, usually referred to as aromatic plants, and hence the aromatic essential oils. These aromatic classes of compounds are abundant in oils of cloves, thyme, wintergreen, etc. The aromatic volatile compounds of essential oils (ACEO) are used in the treatment of several disorders, including infection, inflammation, irritable bowel syndrome (IBS), and as antihelminthics, among others. The ACEO are used in confectionary, beauty products, food, pharmaceuticals, and other medicaments. A plentiful number of over‐the‐counter (OTC) products are also prepared by ACEO and are abundantly distributed in the market. The present review deliberates on the origins, biogenesis, chemical structures, and bioactivities of the ingrained aromatic constituents of the essential oils sourced from different plant species. The review also highlights the importance of ACEO in modern food, cosmetics, and pharmaceutical industries. An overview of the clinical trials of the aromatic constituents and their applications in aromatherapy domains is also outlined. The review addresses a gap in the current literature concerning the aromatic components of essential oils by utilizing prior reviews and research articles. This review serves as a comprehensive source, detailing the chemistry, bioactivity, and therapeutic applications of the aromatic constituents of essential oils, along with their uses in both traditional and contemporary medicine, as well as the mechanistic aspects of the biological activity of these constituents derived from various plant‐sourced essential oils.

## Graphical abstract

The review explores the origins, chemical structures, and bioactivities of aromatic volatile compounds in essential oils from various plants. It highlights their therapeutic uses in treating disorders like infections and IBS, as well as their applications in food, cosmetics, and pharmaceuticals. Additionally, it addresses gaps in the existing literature and provides insights into clinical trials related to these compounds, emphasizing their significance in both traditional and modern medicine.

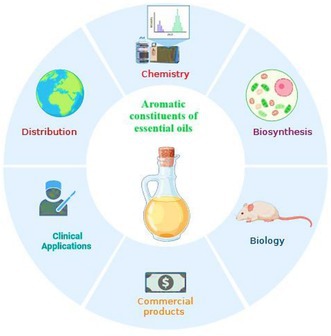

## 1 Introduction

A large number of vastly abundant plant species produce essential oils, one of the major classes of natural products for a variety of purposes, including attracting pollinators, repelling predators, inducing specific physiological functions, such as growth regulation, metabolite detoxification, and roles of plants‐antibiotics, phytoalexins (Arshad et al. 2014; Hoffmann 2020; War et al. 2020). Chemists and other investigators have chemically classified the essential oil compounds in a variety of ways. From a standpoint of chemical structures, they are classified into mono‐, di‐, and sesquiterpenes, based on the number of carbon atoms in their molecular framework. These plant products have also been classified based on their presence of different chemically reactive functional groups, thereby dividing them into hydrocarbons, which are solely composed of carbon and hydrogen atoms, oxygenated hydrocarbons, acid and ester groups containing hydrocarbons as fatty acids and esters, and sulfur and nitrogen‐containing compounds of essential oils. The researchers have also applied an additional structural classification of the volatile oils constituents and the oil itself based on the aromatic character, or aromaticity (saturation and unsaturation of the cyclic volatile compounds) of the ingrained natural constituents into aliphatic and aromatic compounds and aliphatic compounds loaded, or aromatic compounds constituted oils, or simply the aromatic volatile oils (Başer and Demirci 2007; Clarke 2009; Mohammed, Sulaiman, Khan, Al‐Saffar, et al. 2024).

The structural variations and abundance of essential oils in common aromatic plants yield a multitude of commercial and biological benefits (Carrubba and Catalano 2009; Mohammed, Aspatwar, et al. 2024; Mohammed et al. 2023; Sharifi‐Rad, Salehi, et al. 2017; Sharmeen et al. 2021). These oils are also the most common class of natural products in abundance in plants and are used in folklore applications as well as traditional medicines (Mohammed, Al‐Omar, et al. 2019; Zhang et al. 2021). These essential oils are widely known and established for their multi‐pronged involvement in modern industries, mostly based on their contents of aromatic compounds and derivatives thereof. They are a key component of perfumes and cosmetic preparations, play a significant role in the food and flavor industry, and are considered an important natural class of compounds in several pharmaceutical preparations and drug production as starting raw materials, synthons, and intermediates (Irshad et al. 2020; Mohammed et al. 2020; Shaaban et al. 2012). Also, a large population all over the world traditionally used the aromatic essential oils and plants containing the aromatics as digestives, preservatives, and flavoring agents in their foods and as medications for treating minor gastrointestinal, wound‐healing, pain, arthritis, skin conditioner, refresher, and respiratory conditions (Ali et al. 2015; Mohammed, Sulaiman, Khan, Amin, et al. 2024; Mohammed et al. 2021). There is widespread traditional knowledge about the therapeutic benefits and the potential of essential oils, both containing aliphatic and aromatic constituents. A plethora of reports of their use in aromatherapy, Ayurveda, and traditional medical systems like Japanese, Korean, and Chinese are available (Buckle 2014; HALCóN and Buckle 2014; Rhind 2012; Xu et al. 2008). Notwithstanding, both the aliphatic and aromatic volatile compounds that are present together in the distillate of various plant parts as the essential oil components are responsible for the oils' various levels and types of bioactivities. However, the ACEO (aromatic constituents of essential oil) is less prevalent than the aliphatic volatile compounds (AVCs) in most of the aromatic plants—plants that contain larger proportions of aromatic compounds that bear pleasant odors. The aromatic constituent (s) play a crucial role in the odorant properties of these plants, prized for flavor and fragrance, and supposedly responsible for a range of various biological activities (Başer and Demirci 2007). Interestingly, for a few known cases, the ACEO makes up the major proportions of the constituents of essential oils. Owing to these aromatic constituents, the plants have a distinct scent (Cuchet et al. 2019; Jirovetz et al. 2006). The clove has the dominating smell of eugenol, which is the major instilled constituent of the clove essential oil with its percentage ranging between 60% and 900% (Pavithra 2014). Furthermore, in certain other aromatic plants, such as wintergreen and vanilla pods, the single ACEO constituent accounts for the entirety of the plant's essential oils and its chemically and aesthetically aromatic character (Davis 2007; Jadhav et al. 2009).

A majority of ACEOs are generally used in contemporary medicine for the management of several health disorders and diseases. Eugenol, the widespread aromatic constituent of EO, is used as a local anesthetic for toothaches, while the clove pods' essential oil, rich in eugenol, is a primary ingredient in the preparation of toothpaste and mouth gargles (Grogan 2021; Rani et al. 2022). Methyl salicylate, the major product of the American wintergreen ( *Gaultheria procumbens* ) *oil*, is used in the treatment of rheumatic arthritis conditions to control the pain and as a counterirritant (Singewar et al. 2021; Yeoh and Goh 2021). Methyl salicylate also forms a part of the famed volatile mixture, vapor rub, which is used for muscle and joint pain relief, and it serves as a decongestant for cold conditions (Darrell 2022; Smith and Matthews 2022). The current review focuses on the ACEO as an important class of essential oil constituent(s) and the aromatic plants' secondary metabolite(s) roles in human health. The structural aspects, biological activity, clinical applications, therapeutic value, folklore, and traditional uses, in conjunction with the ACEO and aromatic plant occurrence and availability, are discussed.

## 2 Distribution of Aromatic Volatile Oils

The literature overview indicated that the ACEOs have lowered levels of proportional distribution in aromatic plants, as compared to the aliphatic constituents in the volatile oils. Nonetheless, the ACEOs are widely distributed in several plant families, including Apiaceae (Umbellifera), Ericaceae, Styracaceae, Orchidaceae, Myrtaceae, Lamiaceae (Lamiaceae), and Rosaceae families. Specific ACEOs such as *p*‐cymene, carvacrol, and thymol are part of the ingredients of several volatile oils obtained from Origanum, Thymus, Satureja, and Rosa species (Başer and Demirci 2007). In certain plants, across the genera, the aromatic constituents in the volatile oils constitute the majority of the constituents, i.e., eugenol in clove oil (Bhuiyan et al. 2010), thymol in thyme oil (Schwarz et al. 1996), anethole in anise, star anise, and fennel oils (Singh et al. 2006; Yu et al. 2021; Zachariah and Leela 2006), as well as myristicin in the nutmeg, *Myristica fragrans* (Piras et al. 2012). Furthermore, some other plants, such as American wintergreen and almond plants, have single major aromatic constituents in their respective volatile oils (Kwak et al. 2015; Ojha et al. 2022). A summary of the aromatic volatile compounds, their distribution, the plant organs used to obtain the oils, and the oils major traditional uses are summarized (Table 1).

**TABLE 1:** Summary of aromatic structural‐based essential oils (ACEOs) distribution, plant organs used, and major traditional applications.

Aromatic oil	Plant			Organ	Conc.	Major traditional uses	References
	Common name	Latin name	Family				
Methyl salicylate	Wintergreen	*Gaultheria procumbens*	Ericaceae	Leaves		Food salad Defense against pathogens or predators Low back pain relief	Hebert et al. (2014), Knudsen and Tollsten (1993), Rana (2016)
	Sweet and black birch.	*Betula lenta*	Betulaceae	Bark			
Diosphenol	Buchu	*Agathosma betulina*	Rutaceae	Leaves	2.5%	Spasmolytic	Brendler and Abdel‐Tawab (2022)
Thymol	Thyme	*Thymus vulgaris*	Lamiacaeae	Herb	39.5%	Cure problems like indigestion, diarrhea, stomach pain, and fevers. Its components and antibacterial and insecticidal properties	Eftekhari et al. (2019), Escobar et al. (2020), Sarfaraz et al. (2023)
	Basil	*Ocimum gratissimum*					
	Origanum or wild marjoram	*Origanum vulgare*			41.6%–81.1%		
	Wild bergamot or bee palm	*Monarda fistulosa*					
	Ajwain	*Trachyspermum ammi*	Apiaceae	Leaves & Seed	17.41%		
	Moshkoorak or Denak	*Oliveria decumbens*		Flowering aerial parts	38.79%		
Benzyl alcohol	Jasmine	*Jasminum sambac*	Oleaceae	Flowers	7.7%–8.4%	Flowers have historically been used to cure a wide range of illnesses, including toothaches, diarrhea, insomnia, fever, and anxiety	Hattori et al. (1978), Kunhachan et al. (2012), Rahmatullah et al. (2019), Tayoub et al. (2006), Zhu et al. (2021)
	Gardenia	*Gardenia jasminoides*	Rubiaceae		< 3%		
	Ylang‐ylang	*Cananga odorata*	Annonaceae	Tree			
	Tuberose	*Polianthes tuberosa*	Asparagaceae	Flowers	0.09%–0.17%		
	Styrax	*Styrax officinalis*	Styracaceae	Resin	0.6%		
	Lilac	*Syringa pubescens*	Oleaceae	Flower	(3.17)^a^ a: 1 × 10^6^ (area account)		
Safrole	Sassafras	*Sassafras albidum*	Lauraceae	Roots & barks		Beverage and scent in soap and perfume	Saputri et al. (2014), Shibamoto and Bjeldanes (1993), Stubbs et al. (2004)
	Camphor tree	*Cinnamomum camphora*		Leaf, bark & wood	11%–55.4%		
	Nutmeg	*Myristica fragrans*	Myristicaceae	Seed	4.057 (μg/mL)		
Chamazulene	German Chamomile	*Matricaria chamomilla*	Astereaceae	Flower head	2.3%–10.9%	Culinary sector, cosmetics, aromatherapy, and perfumes.	Singh et al. (2011), Ettakifi et al. (2023)
	Roman Chamomile	*Chamaemelum nobile*					
Guaiazulene	German Chamomile	*Matricaria chamomilla*	Astereaceae	Flower head	10.57%		El Mihyaoui et al. (2022), Ettakifi et al. (2023)
	Blue Tansy	*Tanacetum annuum*			4.24%		
Anethole	Anise	*Pimpinella anisum*	Apiaceae	Seed & fruits	80%–90%	Oil is used in flatulence and insomnia treatment, expectorant in cough and flu Herb in culinary purpose	Dobravalskytė et al. (2013), Ekiert et al. (2021), Marinov and Valcheva‐Kuzmanova (2015), Rubab et al. (2017)
	Fennel	*Foeniculum vulgare*			80%		
	Star anise	*Illicium verum*	Schisandraceae	Pods	> 90%		
	Sweet Cicely	*Myrrhis odorata*	Apiaceae	Herb	48%–50%		
	Tarragon	*Artemisia dracunculus*	Astereaceae	Leaf & Herb			
	Basil	*Ocimum basilicum*	Lamiaceae	Leaves	2.4%		
Vanillin	Vanilla orchid	*Vanilla planifolia*	Orchidaceae	Pods		Flavoring ice cream.	Srinivasan (2016)
Eugenol	Clove	*Syzygium aromaticum*	Myrtaceae	Buds	9381.70–14,650 mg/100 g	Headache treatment, spice, and perfumes	Ashokkumar et al. (2022), Petrisor et al. (2022), Ríos and Andújar (2020), Xue et al. (2022), Zachariah and Leela (2006)
	Cinnamon	*Cinnamomum cassia*	Lauraceae	Bark			
	Sweet Basil	*Ocimum basilicum*	Lamiaceae	Leaves	126 mg/g		
	Nutmeg	*Myristica fragrans*	Myristicaceae	Seeds	16.6%		
	Bay	*Laurus nobilis*	Lauraceae	Leaves			
	Lemon Balm	*Melissa officinalis*	Lamiaceae	Leaves	0.5%–28%		
Para cymene	Cumin	*Cuminum cyminum*	Apiaceae	Seeds	23%–39%	Culinary purposes such as vital energy, boosting digestion, and common cold treatment	Almas et al. (2021), Awan et al. (2019), Li et al. (2023), Orav et al. (2003), Ravi et al. (2013)
	Dill	*Anethum graveolens*			6.5%–7%		
	Thyme	*Thymus vulgaris*	Lamiaceae	Herb			
	Marjoram	*Origanum majorana*					
	Eucalyptus	*Eucalyptus globulus, Eucalyptus maculata*	Myrtaceae	Herb	10.10%		
Myristicin	Nutmeg	*Myristica fragrans*	Myristicaceae	Seeds	16.2%	Household spice for GIT, urinary tract disorders, skin diseases, kidney stones, hemorrhoids.	Boutsika et al. (2021), Nikolic et al. (2021), Rema and Krishnamoorthy (2012)
	Parsley	*Petroselinum crispum*	Apiaceae	Leaves	0.51%–44.4%		
	Dill	*Anethum graveolens*		Seeds			
Carvacrol	Origanum or wild marjoram	*Origanum vulgare*	Lamiaceae	Herb	1.6%	Cure problems like indigestion, diarrhea, stomach pain, and fevers. Its components and antibacterial and insecticidal properties	Chávez‐González et al. (2016), Gavaric et al. (2015), Papadatou et al. (2015), Sarfaraz et al. (2023)
	Thyme	*Thymus vulgaris*			12%		
	Wild bergamot or bee palm	*Monarda fistulosa*					
	Winter savory	*Satureja montana*			51.4%–61%		
Styrene	Liquidamber or Sweetgum tree	*Liquidambar styraciflua*	Altingiaceae	Resin	30.9%	Fuel industry	Russo and Russo (1987), Steele et al. (1994)
Cinnamyl alcohol	Camphor tree	*Cinnamomum camphora*	Lauraceae	Leaf, bark & wood		Perfumery and as a deodorant, Beverage and a scent in soap and perfume. Resin used in cough preparations	Duke (2008), Stubbs et al. (2004), de Groot et al. (2025)
	Tolu Balsam	*Myroxylon balsamum*	Papilionaceae	Bark			
	Peru Balsam	*Myroxylon pereirae*	Papilionaceae	Bark	8.08%–24.96%		
Chavicol	Sweet Basil	*Ocimum basilicum*	Lamiaceae	Leaves		Oil in Aromatherapy and culinary purposes as spice on salad and food and relief GIT disorders	Hassanzadeh et al. (2016), Verma et al. (2010), Xue et al. (2022)
	Holy Basil (Tulsi)	*Ocimum sanctum*					
	Tarragon	*Artemisia dracunculus*	Asteraceae	Aerial parts			
	Clove	*Syzygium aromaticum*	Myrtaceae	Buds			
Methyl chavicol	Basil	*Ocimum basilicum*	Lamiaceae	Leaves	68.3%	Oil is used in flatulence and insomnia treatment, expectorant in cough and flu—Herb in culinary purpose	Hassanzadeh et al. (2016), Naaz et al. (2022), Rubab et al. (2017), Vani et al. (2009)
	Tarragon	*Artemisia dracunculus*	Asteraceae	Aerial parts	71.3%		
	Star Anise	*Illicium verum*	Schisandraceae	Pods			
	Fennel	*Foeniculum vulgare*	Apiaceae	Seed & fruits			
Cinnamaldehyde	Cinnamon	*Cinnamomum cassia*	Lauraceae	Bark	83.3%	Headache treatment, spice as flavoring. Beverage and scent in soap and perfume.	Jardim et al. (2018), Stubbs et al. (2004), Zachariah and Leela (2006)
	Camphor tree	*Cinnamomum camphora*		Leaf, bark & wood			
Benzaldehyde	Bitter Almond	*Prunus dulcis*	Rosaceae	Kernel	730.61–17,995 (ng/g)	Oil is used in perfumes and flavorings, it is used to treat rheumatoid arthritis, amenorrhea, dysentery, and diarrhea	Luo et al. (2017), Verma et al. (2017)
	Apricots	*Prunus armeniaca*					
	Peaches	*Prunus persica*			63.1%–98.3%		
	Tolu Balsam	*Myroxylon balsamum*	Papilionaceae	Bark			
Benzyl salicylate	Ylang‐ylang	*Cananga odorata*	Annonaceae	Flower	10.34%	Aromatherapy	Ibrahim (2016), Tan et al. (2015)
	Grenadine or clove pink	*Dianthus caryophyllus*	Caryophyllaceae		14.12%		

## 3 Chemistry of the Aromatic Volatile Compounds From Essential Oils (ACEOs)

**FIGURE 1:**
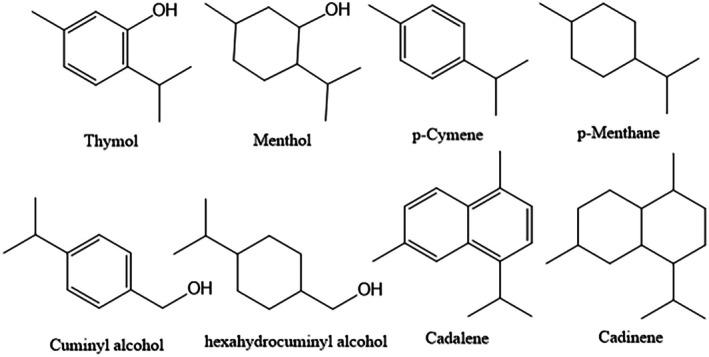
Aromatic and aliphatic analogues present in volatile oils with the corresponding and complementary structural entities.

ACEOs are structurally simple, aromatic, and low‐molecular‐weight compounds that are volatile substances. For the most part, these compounds are benzene ring‐based structures with aliphatic appendages as the extended structure. The thymol and menthol, *p*‐cymene and p‐menthane, cuminyl alcohol and hexahydrocuminyl alcohol, cadalene, and cadinenes, all are aromatic in their structural nature with aliphatic moieties‐based volatile oil analogues (Figure 1) (Başer and Demirci 2007).

**FIGURE 2:**
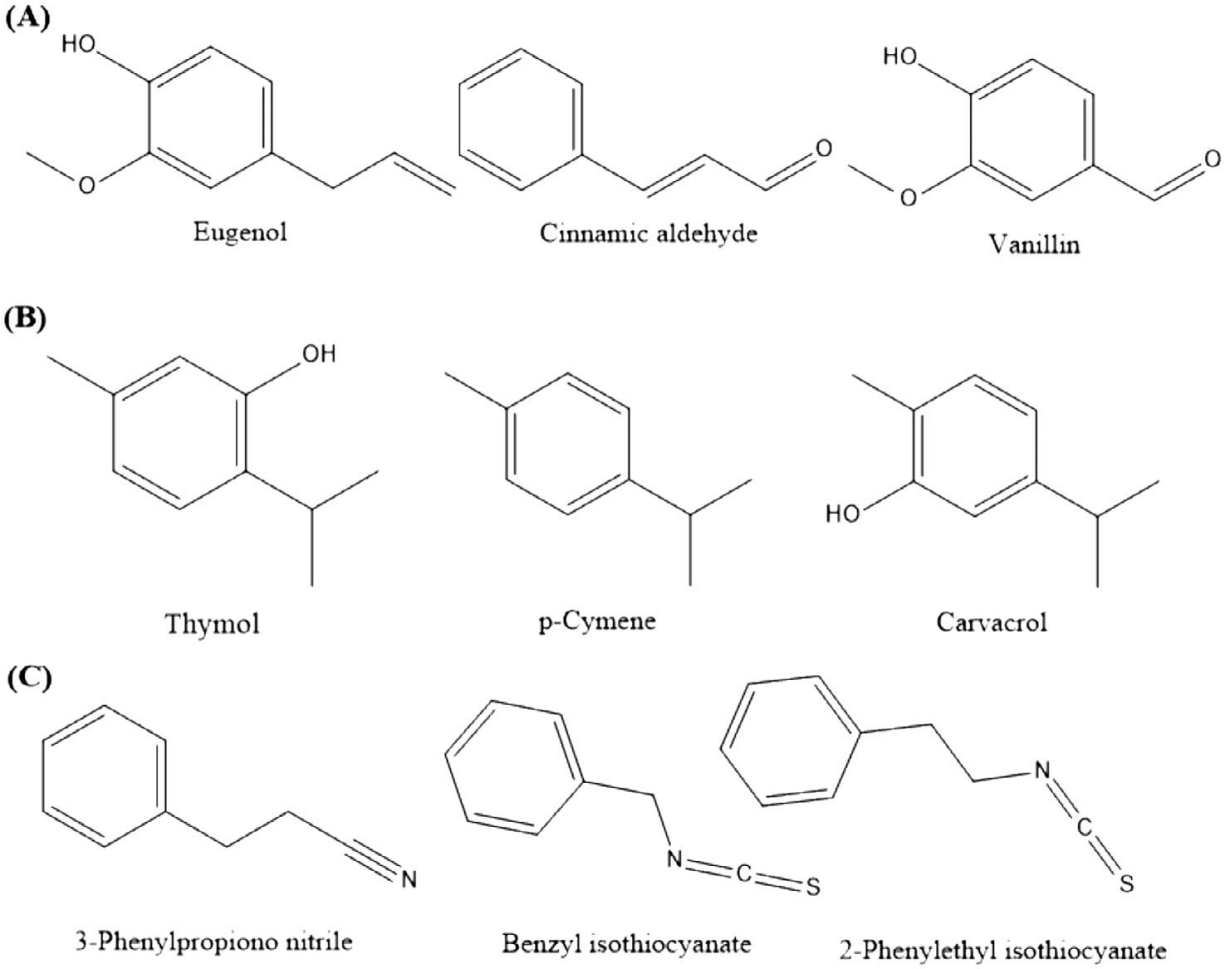
Outlines of the ACEOs constituents biogenic pathways: (A) Phenylpropanoids, (B) terpenoids‐based, with the (C) naturally occurring nitrogen and sulfur‐containing ACEOs.

The ACEOs can be largely classified as aromatic hydrocarbons, and representative compounds cover *p*‐cymene and cadinene, and oxygenated aromatic hydrocarbons, such as thymol and cuminyl alcohol, which are dependent upon the presence or absence of oxygen atom(s)‐based functional group(s) in their structures (Figure 1) (Adam et al. 2011; Voda et al. 2003). The carbon counts based on monoterpenoid, with 10 carbons, such as thymol and *p*‐cymene, and the sesquiterpenoid, consisting of 15 carbons, such as cadalene (Figure 1) (Başer and Demirci 2007), are a common occurrence. Furthermore, the biogenetic pathways of the aromatic volatile oils are also varied; hence, they may be classified as terpenoid or phenylpropanoid ACEOs (Moghaddam and Mehdizadeh 2017; Zuzarte and Salgueiro 2015) (Figure 2). Both phenylpropanoid and terpenoid groups are considered responses to abiotic and biotic plant infections (Korkina 2007; Tholl 2015). Recently, they were reported as rich sources for both the pharmaceutical and cosmetic industries because of their wide variety of pharmacological potential. Regarding the pharmaceutical field, they act as antioxidant, antiviral, antibacterial, antidiabetic, cardioprotective, anticancer, and anti‐inflammatory agents. In cosmetics markets, they are used in wound healing and UV screen creams (Zhu et al. 2024). The biological relevance of terpenoids is recorded by Wagner and Elmadfa; they studied the various activities of terpenoids as antimicrobial, anticancer, and cardiovascular protective abilities (Wagner and Elmadfa 2003). Also, terpenoids are used as natural safe preservatives in the food and candy industries due to their antioxidant powers (Gutiérrez‐del‐Río et al. 2021). Furthermore, nitrogen and sulfur‐containing aromatic volatile oil compounds have also been identified in several plant species. For example, 3‐phenylpropiononitrile, benzyl isothiocyanate, and 2‐phenylethyl isothiocyanate have been identified in the leaves of *Brassica rapa* and seem to generate the characteristic aroma of the plant (Miyazawa et al. 2005). Also, phenethyl isothiocyanate, aromatic and sulfur‐based, has been identified in the canola oil obtained from *Brassica napus* (Abraham and Deman 1985) (Figure 2).

## 4 Biosynthesis of the Aromatic Structural‐Based Essential Oils (ACEOs)

**FIGURE 3:**
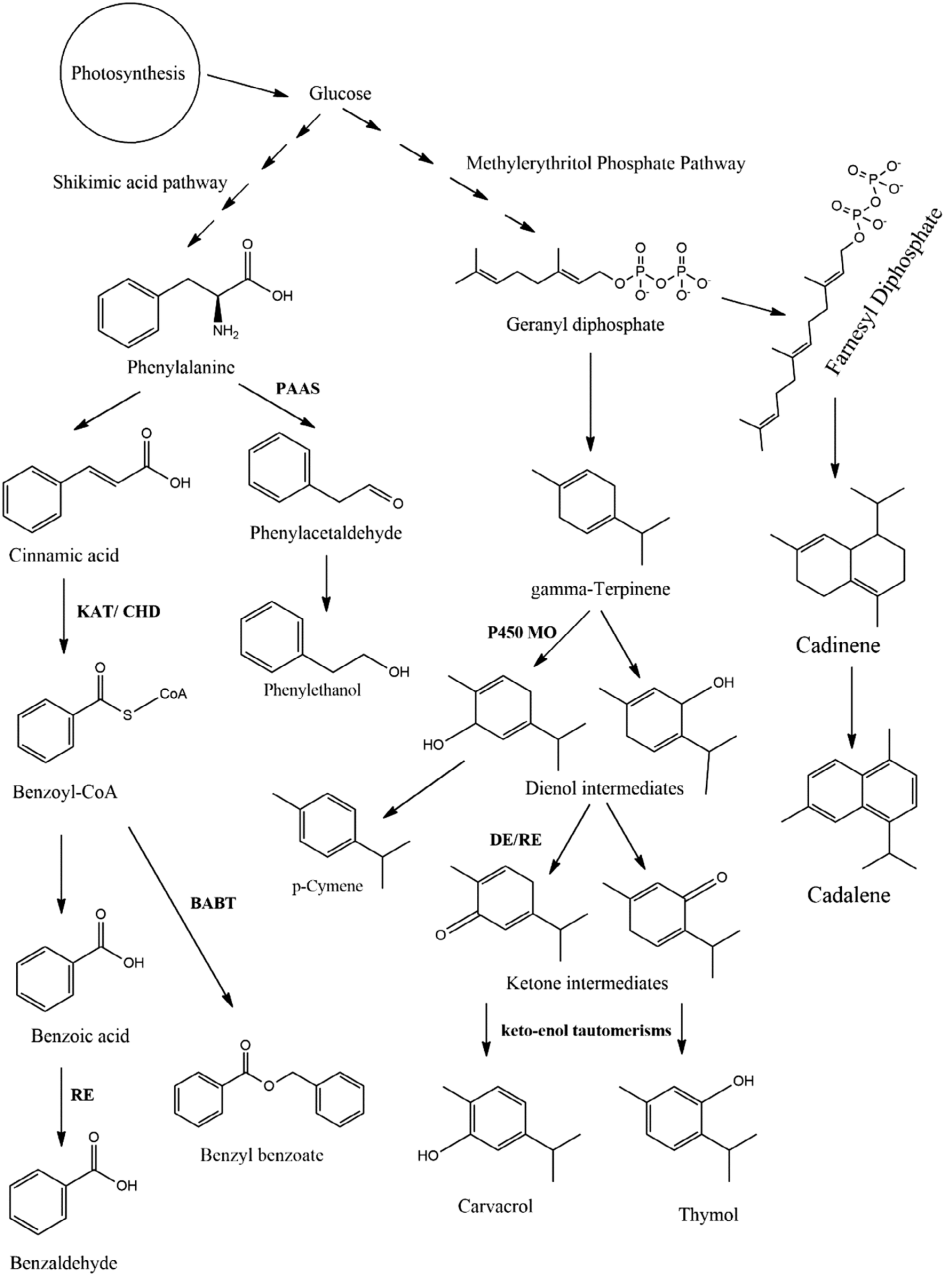
Proposed schematic for biosynthetic pathways: PAAS, phenylacetaldehyde synthase; P450 MO, cytochrome P450 monooxygenases; DE, dehydrogenase; RE, reductase; KAT, 3‐ketoacyl thiolase; CHD, cinnamoyl‐CoA hydratase/dehydrogenase; BABT, benzoyl‐CoA:Salicyl alcohol *O*‐benzoyltransferase.

Based on the chemistry of ACEO, two main biosynthetic pathways have been identified, which are the phenylpropanoid and terpenoid approach. The ACEO biogenetic pathways in plants differ, and most of the ACEO's natural products are derived from the phenylpropanoids (C_6_‐C_3_) approach, which serves the plants as anti‐predators and pollinator attractant materials (Granell and Rambla 2013). This pathway originates from shikimic acid, passes through the degradation of amino acids, and leads to the biosynthesis of several aromatic oxygenated compounds, such as aromatic alcohols, aromatic aldehydes, aromatic ketones, aromatic acids, and aromatic esters (Schwab et al. 2008) (Figure 3). These and other benzenoid volatile constituents are formed from the phenylalanine amino acid template through shortening of the propionic acid sidechain from two carbons (Klempien et al. 2012; Schwab et al. 2008). Generally, the β‐oxidative and non‐oxidative approaches have been defined for the process of 2‐carbon shortening of phenylalanine to produce the benzenoids. The β‐oxidative pathway involves the formation of cinnamoyl‐CoA through the intermediacy of the group of 4‐coumarate: CoA‐ligase (4CL) enzymes (Klempien et al. 2012). In addition, the formation of benzoic acid through the β‐oxidative pathway from phenylalanine via the formation of benzoyl‐CoA has been suggested by Ribnicky et al. (1998), who used isotope‐labeled precursors in *Nicotiana tabacum* . The non‐oxidative‐based benzenoid biosynthetic pathway has also been suggested through the conversion of cinnamic acid to benzaldehyde, the process that resulted in benzoic acid and benzoyl‐CoA‐mediated biosynthesis (Beuerle and Pichersky 2002). The biosynthetic pathway for phenylalanine‐derived essential oils, such as phenylethanol, the major flavor compound in several plants, including rose and tomato, has been described by Tieman et al. through a family of decarboxylases (LeAADC1A, LeAADC1B, and LeAADC2) enzymes (Tieman et al. 2006) starting from the phenylalanine substrate. Tieman et al. have also proposed this pathway for the biosynthesis of 2‐phenylacetaldehyde and 2‐phenylethanol (Tieman et al. 2006). The enzymes involved in the biosynthesis of phenylacetaldehyde from phenylalanine in *Petunia hybrida* have been characterized and named phenylacetaldehyde synthase (PAAS), which catalyzes, through the extraordinarily effective coupling, the phenylalanine decarboxylate (decarboxylation) to oxidation, resulting in phenylacetaldehyde biosynthesis (Kaminaga et al. 2006). Furthermore, transamination as an alternative mechanism to decarboxylation has been proposed by Gonda et al. through the characterization of two genes, i.e., CmArAT1 and CmBCAT1, in melons, which exhibited aromatic and branched‐chain amino acid transaminase involvements (Gonda et al. 2010).

The interconversion of the aliphatic monoterpenes into the corresponding aromatic compounds has been studied by Poulose and Croteau in 1978. The study was conducted as an isotopic dilution experiment, which suggested the conversion of γ‐terpinene (the major aliphatic cyclic diene in thyme essential oil) into the non‐oxygenated aromatic compound, *p*‐cymene, which was further hydroxylated to provide thymol (Poulose and Croteau 1978). Their study was also proved by the incubation of the thyme leaf tissue with the y‐(G‐3H) terpinene, which resulted in an increase in the radioactive *p*‐cymene; however, the study showed that the α‐terpinene and terpinene‐4‐ol were not incorporated in the aromatic compounds, *p*‐cymene and thymol (Poulose and Croteau 1978). Recently, Krause et al. have also proposed the biosynthesis of thymol and carvacrol phenolic volatile oil constituents from γ‐terpinene, which, oxidized by the cytochrome P450 monooxygenases (P450s) of the CYP71D subfamily, resulted in producing the cyclohexadienol intermediates. The dehydrogenation of cyclohexadienol intermediates by the dehydrogenase/reductase resulted in the formation of corresponding ketones, which were then converted to the aromatic entities through keto‐enol tautomerisms (Krause et al. 2021). They also found that two P450 subfamilies, i.e., CYP76S and CYP736A, were responsible for the conversion of thymol and carvacrol through hydroxylation to thymohydroquinone when heterologously expressed in yeast and *Nicotiana benthamiana* (Krause et al. 2021). The sesquiterpenoid‐based ACEO was also biosynthesized from aliphatic sesquiterpenoids. For example, the radio‐labeled 2,7‐dihydroxyl cadalene has been produced in cotton seeds, *Gossypium hirsutum* L, upon treating the plant with [^3^H]‐δ‐*cadinene* (Cane 1999).

## 5 Biological Activities of Aromatic Structural‐Based Essential Oils (ACEOs)

ACEOs are comprised of a complex blend of organic compounds, mainly terpenes, terpenoids, and phenylpropanoids (Moghaddam and Mehdizadeh 2017). The diverse biological activities exhibited by essential oils are attributed to their chemical composition (Mhiri et al. 2018). Constituents of ACEOs contain aromatic rings in their chemical structure (Jaafar et al. 2020), where conjugated double bonds allow the delocalization of π electrons, making the molecules more stable (Zochedh et al. 2022). Aromaticity conferred distinctive properties on the compounds, which, as low‐molecular‐weight entities, are often volatile and have a pleasant odor. Common aromatic ring‐containing components in essential oils (EOs) include phenylpropanoids, benzenoids, phenols, and aromatic entity linked‐terpenes (Gounaris 2010).

The diverse biological activities of ACEOs have been extensively reported (Nieto 2017; Sartoratto et al. 2004). The ACEOs exhibit strong antimicrobial effects against a wide spectrum of bacteria, fungi, protozoa, and viruses. The antibacterial and antifungal properties are due to the disruption of bacterial and fungal membranes by the lipophilic components in the ACEOs (Vergis et al. 2015). The ACEOs modulate host immune response and quorum sensing in bacteria (Reichling 2020). They also display antioxidant capacity through free radical scavenging and metal chelation activity (Chrysargyris et al. 2020; Mohammed 2022). Anti‐inflammatory (Sharopov et al. 2015), analgesic (Sarmento‐Neto et al. 2015), anxiolytic (De Sousa et al. 2015), neuroprotective (Ayaz et al. 2017), anticancer (Mohammed et al. 2023), antimicrobial (Mohammed, Abdel‐Aziz, and Hegazy 2019), and wound‐healing effects (Qureshi et al. 2022) have also been reported. These medicinal properties are attributed to the modulation of various molecular targets and signaling pathways, including receptors, enzymes, cytokines, and genes regulating oxidative stress, apoptosis, and cell proliferation.

### 5.1 Methyl Salicylate

The wintergreen oil, rich in methyl salicylate, which is an organic ester containing the aromatic entity as part of the salicylate group, is an important EO (Michel and Olszewska 2024). It is found in many plants, especially wintergreen leaves and flowers. Methyl salicylate exhibits several therapeutic effects primarily attributed to its salicylate group. It acts as an analgesic by inhibiting prostaglandin synthesis, providing relief from muscle and joint pains (Woodbury et al. 2018). Investigators have demonstrated that using OTC (over‐the‐counter) topical analgesic cream containing 20% methyl salicylate on the skin reduces the narrowing of blood vessels caused by cold and increases the heat loss of exercise‐induced hyperthermia (Wang et al. 2022). Methyl salicylate exhibits anti‐inflammatory effects by suppressing inflammatory mediators like cytokines. Methyl salicylate‐rich stem extract of *Gaultheria procumbens* L., a plant with anti‐inflammatory effects, also reduced the release of IL‐1β, IL‐8, and TNF‐α in a dose‐dependent manner (Michel et al. 2019). As a potent antioxidant, it also scavenges free radicals and protects the cells from oxidative damage (Valverde et al. 2015). Methyl salicylate has also demonstrated antimicrobial activity against certain bacteria, fungi, and viruses (Oloyede 2016). Some studies have shown anticancer effects of methyl salicylate by inducing apoptosis in cancer cell lines. However, high doses can be toxic (Das et al. 2022). Overall, the wide range of biological activities exhibited by methyl salicylate is mediated by its salicylate moiety through modulation of multiple targets, providing the characteristic bioactive properties of the wintergreen oil.

### 5.2 Thymol (2‐Isopropyl‐5‐Methylphenol)

Thymol is a natural monoterpene phenolic compound, found in EOs of plants belonging to the family Lamiaceae. Plants having thymol are *Thymus*, *Ocimum*, and *Origanum*. Thymol contains a hydroxyl group attached to an aromatic ring structure. Thymol, and another aromatic constituent, carvacrol, exhibits a wide range of biological activities, primarily attributed to the presence of the phenolic group in their structures. Thymol nanoemulsions were also developed as an antioxidant agent with potent scavenging activity for use in the food industry (Sedaghat Doost et al. 2019). It was also reported that the extract of thymol showed higher DPPH scavenging activity with a 42.2% inhibition rate as compared to the unformulated thymol constituent (Ramos et al. 2014). In addition, a robust antioxidant activity of thymol at 10 μg/mL was detected in LPS‐stimulated macrophages with NO reduction and ROS protection activities (Ranjbaran et al. 2019). Thymol also displayed strong antimicrobial activity against bacteria, fungi, and certain protozoa by disrupting the cell membrane and cell walls. Thymol was assessed for its effectiveness against Gram‐positive *S. aureus* , with antibacterial activity due to its impact on the lipid bilayers of the cytoplasmic membrane and interaction with the bacterial genomic DNA (Wang et al. 2017). Thymol's mode of action against *Salmonella ser*. and *Typhimurium* involved disrupting the bacterial cell membrane, leading to the unregulated release of intracellular substances, like potassium ions (K^+^), an essential ion for bacterial metabolism and survival. Thymol notably decreased nitrous oxide (NO) production and glutathione levels, aiding in the recovery from oxidative stress (Chauhan and Kang 2014). Medicinally, thymol‐based hydrogels were successfully prepared with significant wound‐healing capacity (Jiji et al. 2019). The antifungal activity of ACEOs containing mainly thymol was effective in eliminating pathogenic strains of *Candida* by means of vapor contact (Mandras et al. 2016). While in dentistry, a new dental resin system doped with thymol was developed to fight oral germs. The resinous material's physical and mechanical properties, bonding strength, and antibacterial effectiveness were assessed. The findings demonstrated significant antibacterial properties and satisfactory compatibility with the host cells. Furthermore, this resin also showed qualities similar to the control resin in terms of toughness and fracture resistance for use in dentistry. The antibacterial properties and biofilm prevention capacities were attributed to thymol's high lipophilicity, which allowed it to accumulate in the bacterial membranes and disrupt their growth (Rezaeian et al. 2019).

**FIGURE 4:**
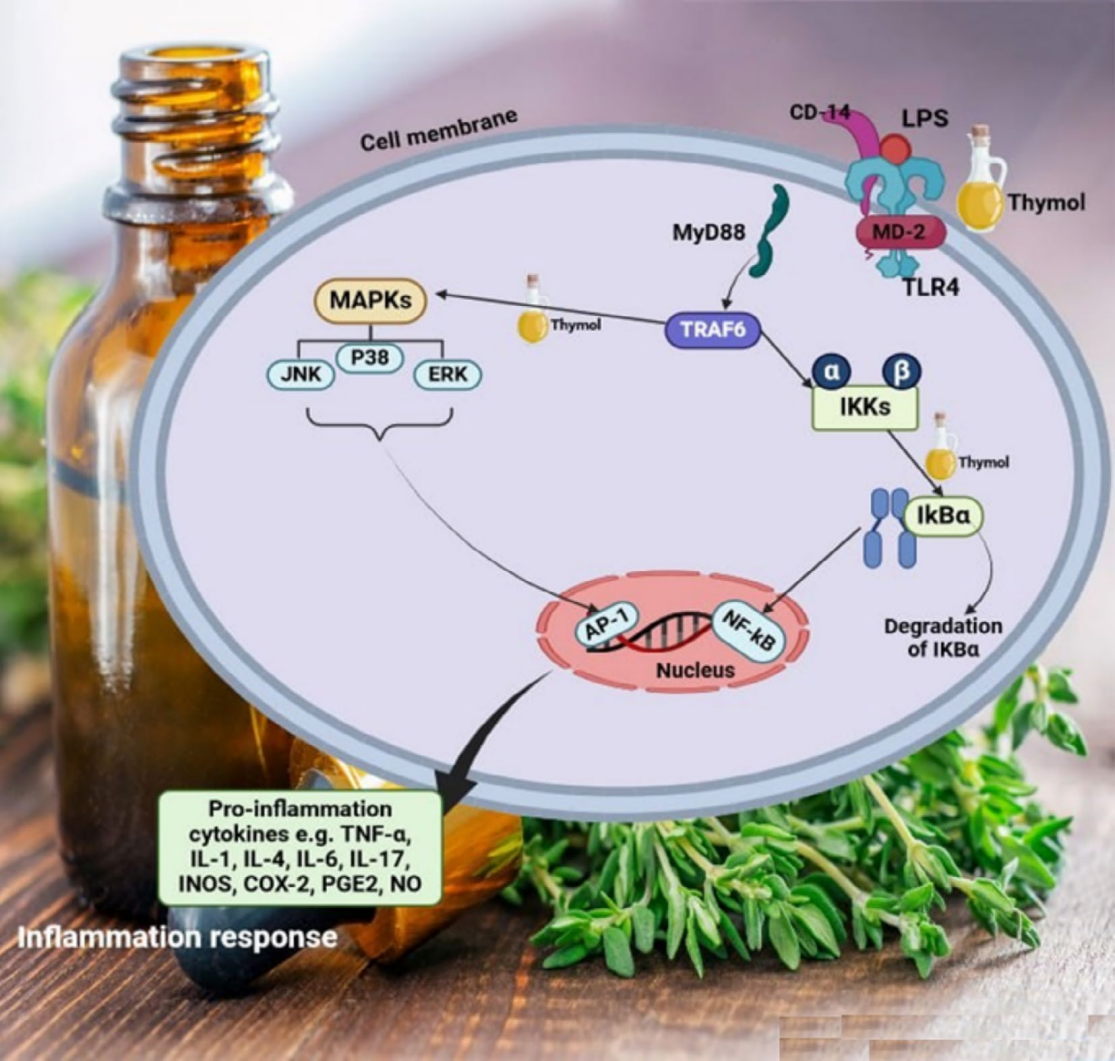
The molecular mechanism of thymol involved in the inflammatory response. Thymol interacts with Toll‐like receptor 4 (TLR4) activated by lipopolysaccharide (LPS), leading to downstream signaling cascade modulation. The compound inhibits the activation of mitogen‐activated protein kinases (MAPKs) including JNK, p38, and ERK, which subsequently prevents the phosphorylation and nuclear translocation of nuclear factor kappa B (NF‐κB). This inhibition results in the suppression of pro‐inflammatory gene transcription and reduced production of inflammatory mediators including tumor necrosis factor‐alpha (TNF‐α), interleukins (IL‐1β, IL‐4, IL‐6, IL‐17), and other cytokines.

Additionally, thymol also demonstrated anti‐inflammatory effects in mouse mammary epithelial cells, which were linked to its ability to inhibit the NF‐κB signaling pathways and p‐38 mitogen‐activated protein kinases (MAPKs) (Figure 4) (Liang et al. 2014). Contrarily, thymol was also found not to suppress the NF‐κB expression, but, nonetheless, exerted anti‐inflammatory effects by inhibiting other pro‐inflammatory transcription factors, like stress‐activated protein kinases SAPK/JNK, signal transducer, and activator of transcription (STAT3), and various other nuclear factors of activated T‐cells (NFATs) (Gholijani et al. 2016). Moreover, experiments on LPS‐stimulated mouse mammary cells showed that treating cells with thymol at a certain concentration range (10–40 μg/mL) caused TNF‐α and IL‐6 suppression, iNOS and COX‐2 expression inhibition, prevention of the activation of p‐38 MAPKs, and other supportive actions (Liang et al. 2014). An asthmatic mouse model orally treated with 4–166 mg/kg of thymol confirmed the decreasing ovalbumin‐specific IgE levels, and production of IL‐4, IL‐5, and IL‐13, thereby preventing the influx of inflammatory cells into the airways (Zhou et al. 2014).

Mechanistically, the central pathway follows thymol interacting with TRAF6, an important signaling protein, which subsequently activates the IKKs (IκB kinases). This activation leads to the degradation of IκBα, an inhibitory protein, allowing the release of NF‐κB, a transcription factor that translocates to the nucleus. Within the nucleus, NF‐κB and AP‐1 (another transcription factor) collaborate to induce the expression of pro‐inflammatory cytokines, such as TNF‐α, IL‐1, IL‐4, IL‐6, IL‐17, iNOS, COX‐2, PGE2, and NO. Additionally, the MAPK (mitogen‐activated protein kinase) cascade, comprising JNK, ERK, and p38, is depicted as being activated by thymol, thereby contributing to the overall inflammatory response (Figure 4).

### 5.3 Carvacrol [2‐Methyl‐5‐(Propan‐2‐yl) Phenol]

**FIGURE 5:**
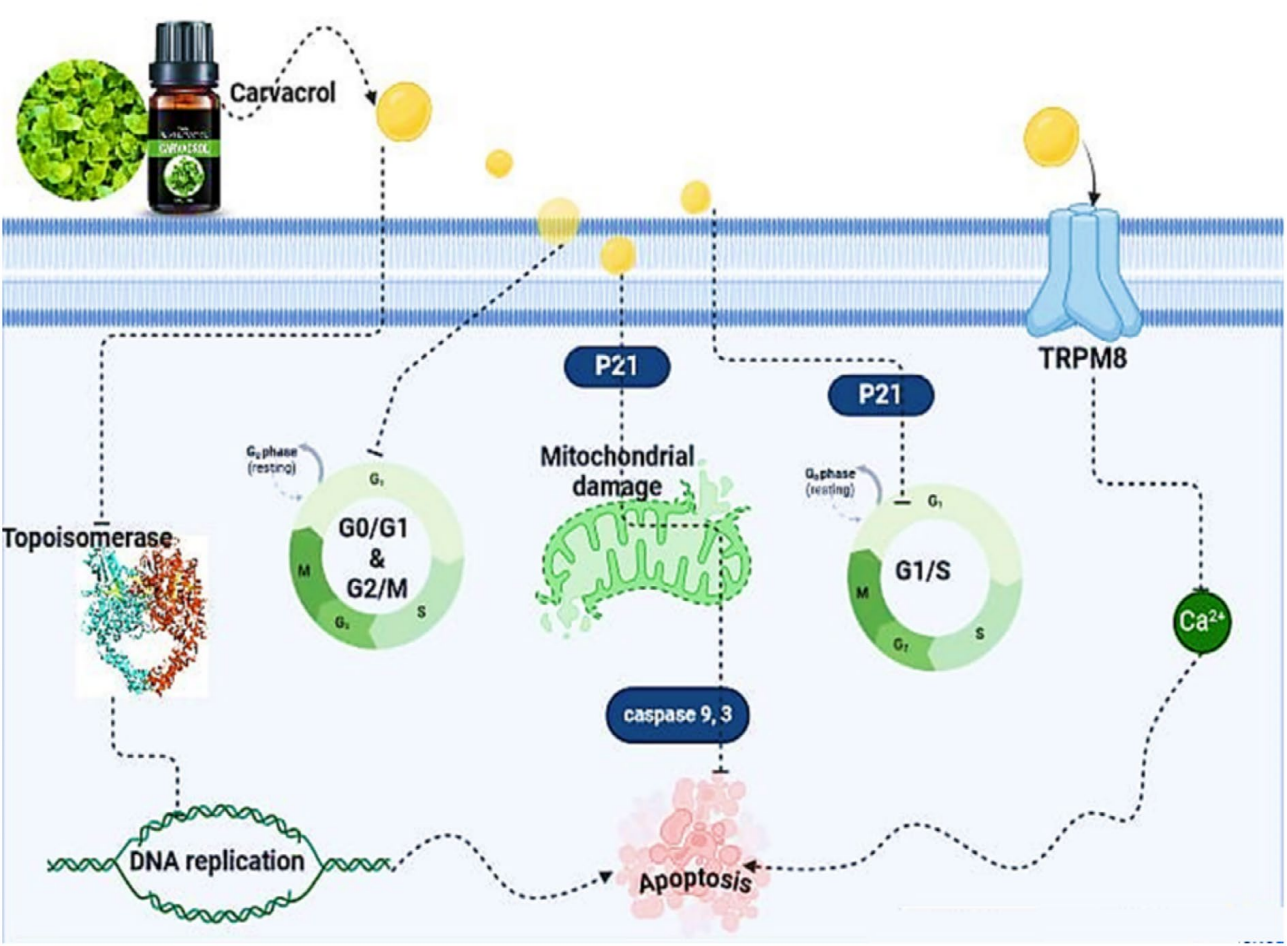
Different mechanisms of carvacrol in apoptosis induction. Carvacrol exposure triggers multiple cellular responses: (1) Cell cycle arrest at G0/G1 and G2/M checkpoints mediated by p21 and p53 tumor suppressor proteins; (2) Mitochondrial dysfunction and damage leading to loss of mitochondrial membrane potential; (3) Activation of TRPM8 (transient receptor potential melastatin 8) channels; (4) Release of cytochrome *c* and activation of caspase‐9 in the intrinsic apoptotic pathway; (5) Induction of topoisomerase‐mediated DNA damage; and (6) Culmination in DNA replication stress and apoptosis.

Carvacrol is a monoterpenic phenol, isomeric with thymol, and is present in various aromatic plants, such as *Origanum dictammus*, *Origanum vulgare* , *Thymbra capitata*, *Thymus vulgaris* , *Thymus serpyllum* , among others (Suntres et al. 2015). Extensive in vitro and in vivo studies exhibited the significance of carvacrol as an antioxidant, anticancer, antimicrobial, anti‐inflammatory, and anti‐obesity agent. Carvacrol also possessed anticancer activity and anti‐growth characteristics (Figure 5). Carvacrol inhibited HepG2 cell growth by inducing apoptosis through the activation of caspase‐3, cleavage of PARP, decreased Bcl‐2 gene expression, and selectively altering the phosphorylation state of the members of the MAPK superfamily. This included decreasing the phosphorylation of ERK1/2 in a dose‐dependent manner, activating the phosphorylation of p38, but not affecting the JNK MAPK phosphorylation (Yin et al. 2012). Carvacrol also dramatically decreased the viability of gastric adenocarcinoma cells at concentrations ranging from 10 to 600 μmol/L (Mari et al. 2021). Carvacrol was used to investigate its effect on cell cycle arrest and apoptosis in breast cancer cell lines. The substance demonstrated anticancer effects by suppressing protein expression, reducing cell growth, and inducing apoptosis in breast cancer cell lines (Li et al. 2021). Carvacrol confirmed its antihypertensive activity by blocking the calcium channels and transient receptors in rat models (Ana Carolina et al. 2020). Because carvacrol can activate the peroxisome proliferator‐activated receptors (PPAR) α and γ, it reduced the expression of cyclooxygenase (COX)‐2 and inhibited the synthesis and activities of nitric oxide (NO). It has also been demonstrated to have anti‐inflammatory properties (Ciavarella et al. 2020).

Carvacrol interacts with TRPM8, a cation channel protein. This interaction leads to the reduction of Bcl‐2 proteins and suppression of p21. The inactivation of p21 and Bcl‐2 causes mitochondrial damage, which in turn activates caspase‐9 and caspase‐3, key enzymes in the apoptotic pathway. Another mechanism involved the inactivation of topoisomerase, followed by DNA damage. Furthermore, carvacrol induced the efflux of Ca^2+^ ions via the activation of the calcium‐permeable channel (TRPM8), resulting in apoptosis (Figure 5).

### 5.4 Safrole

Safrole (4‐allyl‐1,2‐methylene dioxybenzene) is a major chemical constituent of aromatic oils extracted from plants like sassafras, camphor, nutmeg, and black pepper (Bogusz and Al‐Tufail 2008). This colorless or pale‐yellow oil has shown various biological activities. In vivo studies on diabetic rat models showed that oral administration of safrole at doses of 100 and 200 mg/kg improved the diabetic conditions (Rani et al. 2013). Phytochemical analysis revealed that the extract of *Piper auritum* that contained 71.8% safrole showed potent antioxidant activity and inhibition of lipid oxidation (Rodríguez et al. 2013). As an anti‐inflammatory agent, it was reported that safrole effectively caused inhibition of leukotriene, an inflammatory mediator produced by leukocytes, suggesting the use of safrole as an inhibitor against a number of inflammatory diseases, including rhinitis, cystic fibrosis, asthma, and rheumatoid arthritis (Ahn et al. 2001). As an antimicrobial agent, safrole exerted killing activity against a vast number of pathogenic bacteria, including *Enterococcus faecium* , *Pseudomonas aeruginosa* , and *Proteus vulgaris* (Eid and Hawash 2021). Photosynthesized dimeric safrole effectively inhibited the growth of *Candida albicans* (Khayyat 2013). The anticancer activity of safrole was previously reported. The cytotoxic effect of safrole against HL‐60 cells resulted from the induction of apoptosis through reduction of the mitochondrial membrane potential, thereby releasing the cellular reactive oxygen species and triggering the expression of pro‐apoptotic genes (Chun‐Shu et al. 2012). Also, safrole induced apoptosis by upregulation of BAX and Bid genes, together with downregulation of the Bcl‐2 gene in the human tongue squamous carcinoma SCC‐4 cell line (Yu et al. 2012).

### 5.5 Chamazulene

Chamazulene, a naturally occurring sesquiterpene lactone found primarily in the ACEOs of various plant species, notably German chamomile ( *Matricaria recutita* ) and Yarrow ( *Achillea millefolium* ) (Mohammed et al. 2023; Russo et al. 2020). Chamazulene gained significant interest in scientific research due to its diverse biological activities and potential therapeutic applications. Chamazulene is well known as a potent antioxidant agent with significant neutralizing capability of free radicals and inhibition of lipid peroxidation (Michelakis et al. 2016). Capuzzo et al. indicated strong total antioxidant activity of chamazulene in chamomile oil with IC_50_ at 6.4 μg/mL, significantly higher than α‐tocopherol and ascorbic acid. It was reported that chamazulene was unable to react with DHHP; however, a potent scavenging rate was observed when chamazulene was assayed with ABTS (Capuzzo et al. 2014). Chamazulene‐rich EO of *Artemisia arborescens* showed effective scavenging activity of the ABTS radical cation (Ornano et al. 2013). As an anti‐inflammatory agent, chamazulene acted as a hepatoprotective agent against ethanol‐induced injury of the liver by ameliorating the levels of liver's functional enzymes and alleviating the oxidative stress (Wang et al. 2020). The protective effects of chamazulene in osteoarthritis‐induced rats showed significant reduction in TNF‐α and IL‐6‐induced inflammation (Ma et al. 2020). Chamazulene‐rich EO from *A. arborescens* also initiated apoptosis in melanoma A375 cancer cell lines by disrupting the cellular mitochondria and accumulation of ROS (Ma et al. 2020).

### 5.6 Cinnamaldehyde

Cinnamaldehyde is an aromatic flavonoid with a strong flavor and aroma, found in the bark of cinnamon trees, particularly *Cinnamomum verum* and *C. cassia* . Cinnamaldehyde exhibited diverse biological potential and therapeutic applications. It possesses antioxidant (Othman et al. 2020), anticancer (Hong et al. 2016), neuroprotective (Kuru Bektaşoğlu et al. 2021), antidiabetic (Zhu et al. 2017), and cardioprotective effects (Luan et al. 2022). The antimicrobial and anti‐biofilm activities of cinnamaldehyde suggested it as a promising antimicrobial agent against a wide range of pathogenic microbes, i.e., fungi, bacteria, and viruses. Cinnamaldehyde has demonstrated efficacy against both Gram‐positive and Gram‐negative bacterial biofilm growth, including the biofilms produced by *Pseudomonas aeruginosa* and *Staphylococcus aureus* (Topa et al. 2018). Besides its action against *Streptococcus mutans* , cinnamaldehyde also decreased the bacterial biofilm and downregulated the gene expression of various virulent genes associated with biofilm formation (He et al. 2019). Induced deposition of β‐amyloid plaques (major sign of Alzheimer's disease) in SHSY5Y neuronal cells was significantly reversed under in vitro conditions due to increasing concentrations of cinnamaldehyde, thereby confirming its neuroprotective effects against Aβ neurotoxicity (Emamghoreishi et al. 2019). Recently, cinnamaldehyde reduced the harmful effects on the heart and kidneys caused by cyclophosphamide. This was shown by a significant drop in markers of cardiac and renal injury, together with the restoration of normal tissue alterations (Abd El Salam et al. 2023).

### 5.7 Myristicin

Myristicin is a biodynamic naturally occurring aromatic compound that is mainly found in EOs of nutmeg ( *Myristica fragrans* ) seeds (Brixius 2018) and has shown several promising bioactivities. Myristicin from different plant sources effectively enhanced the effectiveness of the antioxidant enzymes, including catalase, superoxide dismutase, glutathione peroxidase, and glutathione reductase. Additionally, myristicin also reduced the levels of lipid peroxidation. Under in vitro conditions, the EOs containing myristicin exhibited significant antioxidant activity (Boulebd 2019; Rahman et al. 2018). Myristicin has the ability to reduce inflammation by blocking several cytokines and inflammatory mediators involved in the chemotaxis of inflammatory processes. These include TNF‐α, interleukins, nitric oxide, macrophage inflammatory proteins (MIP‐1α and MIP‐1β), colony stimulating factor, and myeloperoxidase (Qiburi et al. 2020). As an anticancer agent, the EO of *Myristica fragrans* , which contained 32% myristicin, effectively decreased the viability of human colorectal adenocarcinoma cells (Caco‐2). In addition, the myristicin containing oil demonstrated an IC_50_ value of 146 μg/mL, suggesting that the myristicin might be the active ingredient responsible for the oil's cytotoxic effects (Piras et al. 2012). Myristicin in its pure form also effectively prevented the proliferation of AA8 and EM9 ovarian cell lines, which triggered cell death by activating the caspases (Martins et al. 2011).

### 5.8 Guaiazulene

Guaiazulene, azulene 1,4‐dimethyl‐7‐isopropylazulene, is a bicyclic aromatic sesquiterpene that is a constituent of many plants' EOs, e.g., *Matricaria chamomilla* L., *Guaiacum officinale*, and chamomile oils (Ma et al. 2023). Over the last few decades, researchers have examined guaiazulene to assess the potential pharmacological effects in treating different diseases. Guaiazulene possesses antiseptic, anti‐inflammatory, antibacterial, antioxidant, antimutagenic, immunomodulatory, fungicidal, expectorant, diuretic, diaphoretic, demulcent, and bitter stimulant activities and effects (Akram et al. 2023). Guaiazulene and its derivative (2‐hydroxyazulenes) were found to exhibit antiretroviral activity, effectively inhibiting the replication of HIV‐1 (Peet et al. 2016). Guaiazulene and azulene derivatives were successfully applied in the treatment of gastric ulcers (Najm 2011). Several alterations to the azulene system with putative antiulcer properties have already been conducted. In vivo testing using a mouse model of stomach injury induced by ethanol showed that two guaiazulene derivatives exerted anti‐gastric ulcer activity compared to the positive control Omeprazole (Cao et al. 2016). Guaiazulene has been documented to exhibit antiproliferative properties against various human oral tumor cell lines. Guaiazulene effectively inhibited the proliferation of Caco‐2 cells (Vinholes et al. 2014). The antineoplastic activity of guaiazulene derivatives was assessed on various cancer cell lines (60 different types), and derivatives showed selective inhibition against the cancer cell types (Hong et al. 2001). Guaiazulene was reported to enhance glucose uptake in muscle cells and adipocytes, thereby improving insulin sensitivity (Ikegai et al. 2013). Additionally, guaiazulene has been found to stimulate insulin secretion from pancreatic beta cells, aiding in the regulation of blood glucose levels (Chen et al. 2019). Animal models of diabetes have further validated guaiazulene's antidiabetic activity. Administration of guaiazulene has been shown to reduce blood glucose levels, improve glucose tolerance, and ameliorate insulin resistance in diabetic animals (Akram et al. 2023).

### 5.9 Methyl Chavicol

Methyl chavicol, also known as estragole, or 4‐allylanisole, is an aromatic natural compound composed of a benzene ring substituted with a methoxy and an allyl group. Methyl chavicol is the primary constituent of EO extracted from different plants including pine, fennel, bay, and basil (*Ocimum ciliatum*). Methyl chavicol has been the subject of considerable scientific interest due to its diverse biological activities and potential applications in various fields. Methyl chavicol is known for its well‐documented antibacterial and antifungal effects. Methyl chavicol, as a predominant constituent of basil oil (87.6%), is reported to have bacteriocidal activity against a wide range of phytopathogenic bacteria (Moghaddam et al. 2014). EO of *O. basilicum* L. containing 23.8% methyl chavicol has shown antimicrobial activity against Gram‐positive and Gram‐negative pathogenic bacteria, with the best results reported on coagulase‐positive *Staphylococcus* (Stanojevic et al. 2017). The high percentage of methyl chavicol (93.24%) in *O. selloi* EO was able to inhibit mycelial growth of *Alternaria alternata* , *Colletotrichum gloeosporioides*, and *Moniliophthora perniciosa* (Costa et al. 2015). Methyl chavicol has been documented to have antioxidant characteristics, which can be ascribed to its capacity to eliminate free radicals and impede lipid peroxidation. The inhibition of lipid peroxidation and antioxidant potentials of methyl chavicol were reported to be 73.08% and IC_50_ at 312.5 μg/mL, respectively (Santos et al. 2018). The antioxidant activity of the EO from Egyptian *O. basilicum* L. was found to be different in activity, with an IC_50_ value of 0.21 mg/mL. The antioxidant activity difference was related to the variations in the chemical ingredients of the oils in which methyl chavicol constituted 27.82% of methyl chavicol (Farouk et al. 2016). Recently, the neuroprotective properties of EOs containing methyl chavicol showed the ability to attenuate neuroinflammation, reduce oxidative stress, and protect neural cells from various threats (Abd Rashed et al. 2021). Neuroprotective and antioxidant properties of *O. basilicum* EO in preventing seizures induced in mice by pentylenetetrazole (PTZ) were examined under in vivo conditions. Significantly, *O. basilicum* EO had anticonvulsant properties and protected the brain from oxidative damage caused by the PTZ (Mansouri et al. 2022).

### 5.10 Vanillin

**FIGURE 6:**
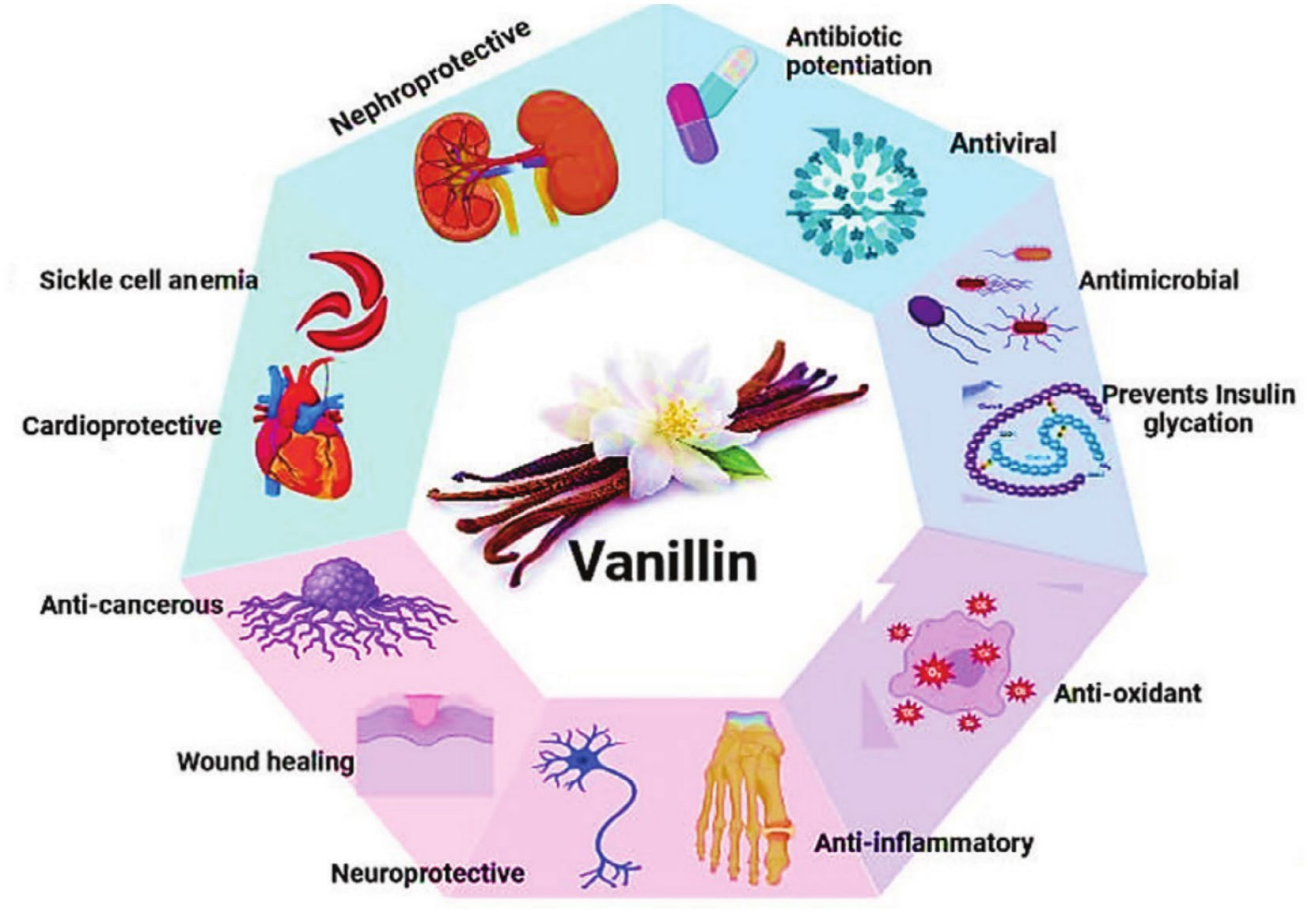
Various biological activities and pharmaceutical significance of vanillin.

Vanillin, 4‐hydroxy‐3‐methoxybenzaldehyde, an aromatic phenolic aldehyde compound that was originally isolated from *Vanilla planifolia* , *V. tahitensis*, and *V. pompona* (Arya et al. 2021), is an important aromatic constituent of volatile oils. Vanillin has long been utilized as a flavoring agent in food and drinks globally. However, recent scientific findings indicated that it possessed several biological properties (Figure 6). Vanillin exhibited antagonistic effects on genotoxic compounds by synergistically enhancing the mitotic recombination and mutation protection (Santos et al. 1999). In the same way, vanillin also showed antimutagenic properties in somatic cells of *Drosophila melanogaster* exposed to different mutagens (Sinigaglia et al. 2004). As an anticancer, vanillin was reported to induce cell cycle arrest during G_2_/M and G_1_/G_0_ phases of HT‐29 cells (Ramadoss and Sivalingam 2020). Complexes derived from vanillin by Schiff base reaction have shown potent anticancer activity against human colorectal cancer HCT116 cell lines (Bahron et al. 2019). Moreover, in vivo study showed that administration of vanillin at doses from 125 to 5000 mg/kg body weight for a period of 3 weeks caused apoptosis and increased the activity of substances that protected against oxidative damages in mice with solid Ehrlich tumors (Elsherbiny et al. 2016).

Vanillin is also crucial in the prevention and management of pathological disorders characterized by high blood sugar levels (hyperglycemia) and high levels of fats in the blood (hyperlipidemia). Vanillin, obtained from *Gastrodia elata*, reduced insulin resistance by decreasing fat formation in adipocytes and promoting lipolysis and leptin signaling in overweight rats (Park et al. 2011). A study by Lu et al. found that vanillin at doses of 100 and 200 mg/kg body weight significantly decreased blood glucose levels by 54% and reduced insulin levels. Additionally, the levels of triacylglycerol and creatinine, as well as the levels of alanine and aspartate aminotransferase activities, and the inflammatory markers like TNF‐α, IL‐6, and IL‐1 β were improved (Lu et al. 2019). Vanillin has also been found to act as a strong scavenger of ROS in various antioxidant tests, such as ORAC, ABTS+, and oxidative hemolysis inhibition. The free radical quenching ability of vanillin is attributed to its self‐dimerization, which leads to high reaction stoichiometry (Tai et al. 2011). Additionally, it has been discovered that vanillin exhibits anti‐inflammatory properties. For example, a study by Lim et al. demonstrated that vanillin inhibits nitric oxide production in lipopolysaccharide‐activated RAW264.7 macrophages. Furthermore, the inhibition of inducible nitric oxide synthase is strongly associated with anti‐inflammatory effects (Jung et al. 2008). Vanillin has also been studied for its hepatoprotective and nephroprotective effects (Saad et al. 2017). The in vivo induced hepatotoxic effect by CCl_4_ in rats was prevented by administering vanillin intraperitoneally at a dose of 150 mg/kg. This was achieved by reducing the activities of serum transaminases and increasing the protein levels (Makni et al. 2011). Additionally, vanillin showed protective effects against renal injury caused by cisplatin and methotrexate. This protection was observed through decreased levels of elevated renal markers, like serum creatinine and cystatin C, and through reducing iNOS, TNF‐α, IL‐18, cytosolic cytochrome C, and caspase‐3 while improving the renal total antioxidant capacity and the Bcl‐2/Bax expression ratio in pretreated rats (Fouad and Al‐Melhim 2017).

### 5.11 *p*‐Cymene

**FIGURE 7:**
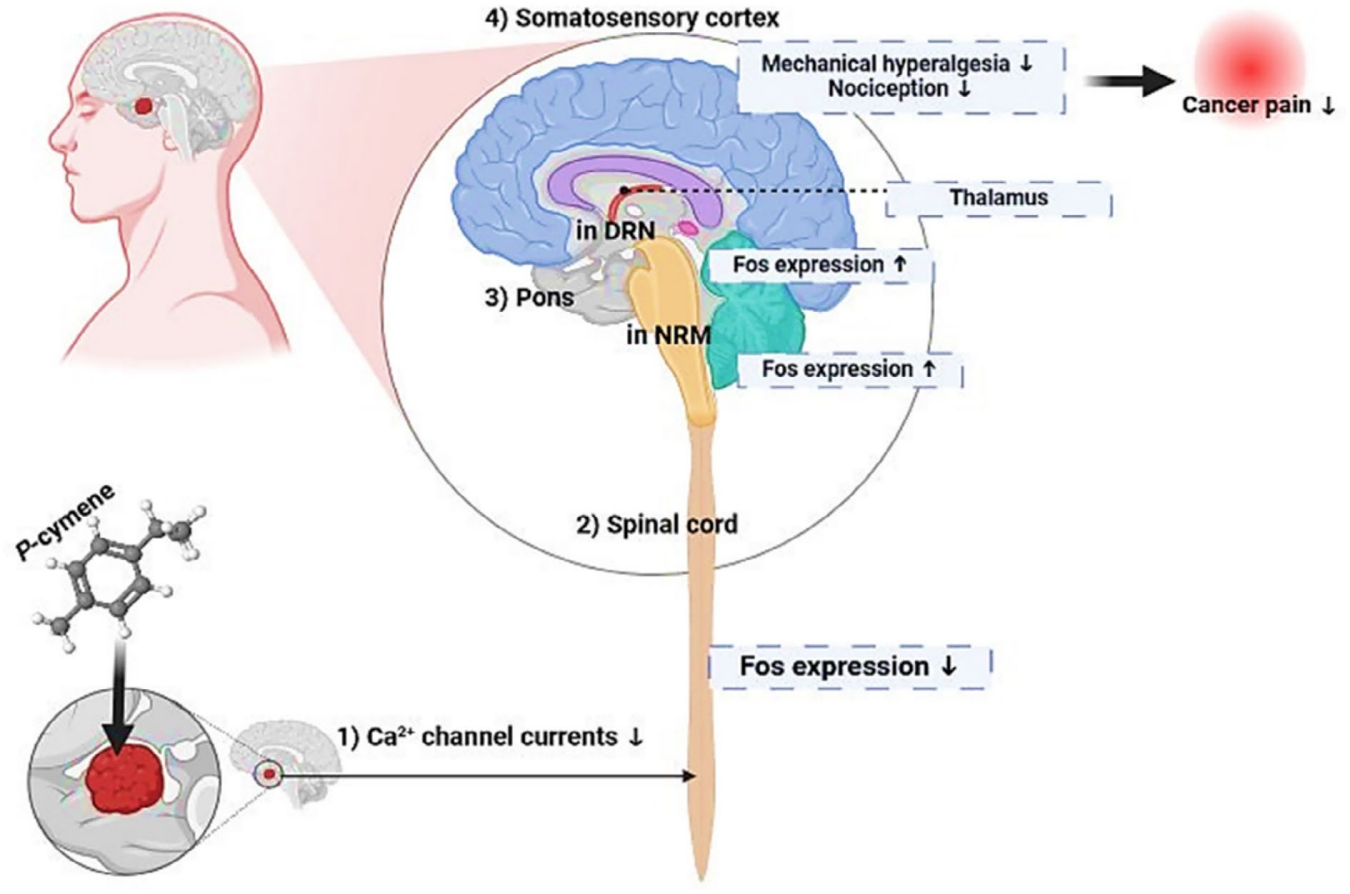
*p*‐Cymene mode of action for reducing cancer‐associated pain. The mechanism involves four key anatomical regions: (1) Peripheral level—Carvacrol reduces Ca^2+^ channel currents in nociceptive neurons, decreasing pain signal initiation; (2) Spinal cord—Downregulation of Fos expression in dorsal horn neurons, indicating reduced pain transmission and central sensitization; (3) Brainstem (Pons)—Enhanced Fos expression in the nucleus raphe magnus (NRM) and dorsal reticular nucleus (DRN), suggesting activation of descending pain inhibitory pathways; (4) Somatosensory cortex—Reduced mechanical hyperalgesia and nociception, along with decreased thalamic pain processing, ultimately resulting in diminished cancer pain perception.

*p*‐Cymene, a naturally occurring alkyl‐isoprenoid aromatic monocyclic monoterpene, is commonly found in EOs of aromatic plants such as *Origanum*, *Ocimum*, *Thymus*, and Eucalyptus. This alkyl‐isoprenoid aromatic compound has shown various pharmacological properties (de Santana et al. 2015). The administration of *p*‐cymene resulted in a significant reduction in nociception behavior in both the hot‐plate test and the acetic acid‐induced writhing test. In a study conducted by Santana et al., the anti‐nociceptive activity of *p*‐cymene demonstrated that *p*‐cymene reduced orofacial nociception pain induced by formalin, capsaicin, and glutamate (Santana et al. 2011). *p*‐Cymene exhibited promising analgesic properties in alleviating cancer‐associated pain by modulating nociceptive pathways and inflammatory mediators, thereby reducing pain perception and hence improving the quality of life for cancer patients who were experiencing chronic pain (Figure 7) (Santos et al. 2019).

The cancer‐related pain reduction involved downregulating Ca^2+^ channel current, modulating the Fos expression in the spinal cord and pons, and attenuating mechanical hyperalgesia as well as nociception in the somatosensory cortex. Subcutaneous administration of *p*‐cymene resulted in decreases in both Ca^2+^ channel currents and the level of Fos expressions in the spinal cord. However, the Fos expression declined in the pons, especially in the periaqueductal gray (PAG) and nucleus raphe magnus (NRM), which attenuated the pain associated with cancer by reducing mechanical hyperalgesia and nociception through the somatosensory cortex (Figure 7).

The anticancer effect of *p*‐cymene was potentially evaluated against several cancer cell lines. The *p*‐cymene effectively inhibited the production of MMP‐9 generated by 12‐O‐tetradecanoyl‐phorbol‐13‐acetate treatment in HT1080 human fibrosarcoma cell lines. This inhibition was achieved by blocking the ERK1/2 and p38 MAPK signaling pathways, which reduced the invasive behavior of the HT‐1080 cells to its lowest levels (Li et al. 2016). Vajs et al. (2015) demonstrated that different *p*‐cymene complexes with ruthenium (II) exerted high cytotoxic action against various cancer cell lines, i.e., HEp‐2 cells and their drug‐resistant HEp‐2 subline (7 T), HCT‐116, H460, and MDA‐MB‐435 cells. In this study, the complexes triggered apoptosis by causing the cells to accumulate in the S phase of the cell cycle, with the accumulation being dependent on both time and dose.

Also, several investigators have reported the antidiabetic effect of *p*‐cymene. Abbasi et al. conducted an in vitro investigation on the anti‐glycation impact of *p*‐cymene using a mix of electrochemical, chemometric, and docking experiments. *p*‐Cymene at a concentration of 2.5 mg/mL effectively suppressed the glycation (Abbasi et al. 2018). A separate study examined the impact of *p*‐cymene in mice models treated with a high‐fat diet. The findings demonstrated that *p*‐cymene had a noteworthy impact on the levels of glucose in the animals blood that were fed a high‐fat diet (Lotfi et al. 2015). As an antiviral agent, *p*‐cymene was reported to exhibit in vitro antiviral activity against herpes simplex virus type 1 by reducing viral replication and plaque formation in Vero cells (Sharifi‐Rad, Sureda, et al. 2017). Molecular simulation on the ability of *p*‐cymene to inhibit RNA viruses showed that *p*‐cymene binds at the lowest binding affinity to the C‐terminal domain that contained a nuclear localization signal. This binding impedes the nuclear translocation of viral protein and restrains the virus proliferation (Panagiotopoulos et al. 2021). Pharmaceutically, *p*‐cymene is notably exploited as an antioxidant agent. It exhibited in vivo antioxidant properties and has the potential to function as a neuroprotective agent in the hippocampus of adult mice by measuring the levels of thiobarbituric acid reactive substances (TBARS), nitric oxide (NO), catalase (CAT), and superoxide dismutase (SOD) activity. Administration of *p*‐cymene led to a notable reduction in lipid peroxidation and nitrite levels (de Oliveira, de Carvalho, et al. 2015).

Table 2 presents a comprehensive overview of twenty major aromatic volatile compounds of EOs, detailing their botanical sources, taxonomic classification, and key biological activities.

**TABLE 2:** Aromatic volatile compounds of essential oils (ACEOs): Source plants, taxonomic classification, and key biological activities.

No.	Aromatic compound	Source plants	Plant family	Key biological activities	Ref.
**1**	Methyl salicylate	Wintergreen ( *Gaultheria procumbens* ), Sweet birch ( *Betula lenta* )	Ericaceae, Betulaceae	Analgesic (pain relief for muscles and joints) Anti‐inflammatory Antioxidant (free radical scavenging) Antimicrobial Vasodilation and heat loss enhancement	Michel and Olszewska (2024), Woodbury et al. (2018)
**2**	Thymol	Thyme ( *Thymus vulgaris* ), Oregano ( *Origanum vulgare* ), Wild bergamot ( *Monarda fistulosa* )	Lamiaceae	Strong antimicrobial (bacteria, fungi, protozoa) Antioxidant (DPPH scavenging, ROS protection) Anti‐inflammatory (inhibits NF‐κB, p38 MAPK) Wound healing Membrane disruption in pathogens	Gabbai‐Armelin et al. (2022), Ranjbaran et al. (2019), Sedaghat Doost et al. (2019)
**3**	Carvacrol	Oregano ( *Origanum vulgare* ), Thyme ( *Thymus vulgaris* ), Winter savory ( *Satureja montana* )	Lamiaceae	Anticancer (apoptosis induction, MAPK modulation) Anti‐inflammatory (PPAR activation, COX‐2 inhibition) Antimicrobial Hypertension control (calcium channel blocking) Antioxidant	Dias et al. (2022), Mari et al. (2021)
**4**	Eugenol	Clove ( *Syzygium aromaticum* ), Cinnamon ( *Cinnamomum cassia* ), Sweet basil ( *Ocimum basilicum* )	Myrtaceae, Lauraceae, Lamiaceae	Local anesthetic (dental applications) Antimicrobial Anti‐inflammatory Antioxidant Pain relief	Cherdchom et al. (2021), Damasceno et al. (2024)
**5**	Vanillin	Vanilla ( *Vanilla planifolia* )	Orchidaceae	Anticancer (cell cycle arrest, apoptosis) Antidiabetic (insulin sensitivity) Anti‐inflammatory Antioxidant (ROS scavenging) Hepatoprotective and nephroprotective	Olatunde et al. (2022), Ramadoss and Sivalingam (2021)
**6**	Anethole	Anise ( *Pimpinella anisum* ), Fennel ( *Foeniculum vulgare* ), Star anise ( *Illicium verum* )	Apiaceae, Schisandraceae	Antispasmodic Expectorant Digestive aid Antimicrobial Flavoring agent	Baky et al. (2020), Lima et al. (2020)
**7**	Safrole	Sassafras ( *Sassafras albidum* ), Camphor tree ( *Cinnamomum camphora* ), Nutmeg ( *Myristica fragrans* )	Lauraceae, Myristicaceae	Antidiabetic Antioxidant Anti‐inflammatory (leukotriene inhibition) Antimicrobial Anticancer (apoptosis induction)	Ahn et al. (2001), Eid and Hawash (2021)
**8**	Chamazulene	German chamomile ( *Matricaria chamomilla* ), Roman chamomile ( *Chamaemelum nobile* )	Asteraceae	Potent antioxidant (free radical scavenging) Anti‐inflammatory Hepatoprotective Anticancer (apoptosis in melanoma cells) Skin soothing properties	Batovska et al. (2025), Gabbanini et al. (2024)
**9**	Guaiazulene	German chamomile ( *Matricaria chamomilla* ), Blue tansy (*Tanacetum annuum*)	Asteraceae	Antiseptic and anti‐inflammatory Antibacterial and antifungal Antiretroviral (HIV‐1 inhibition) Anti‐gastric ulcer Anticancer and antidiabetic	Bakun et al. (2021), Ma et al. (2023)
**10**	*p*‐Cymene	Cumin ( *Cuminum cyminum* ), Thyme ( *Thymus vulgaris* ), Eucalyptus species	Apiaceae, Lamiaceae, Myrtaceae	Anti‐nociceptive (pain reduction) Anticancer (MMP‐9 inhibition, MAPK modulation) Antidiabetic (anti‐glycation) Antiviral (herpes simplex virus) Neuroprotective	Asle‐Rousta and Peirovy (2025), Pyo and Jung (2024)
**11**	Myristicin	Nutmeg ( *Myristica fragrans* ), Parsley ( *Petroselinum crispum* ), Dill ( *Anethum graveolens* )	Myristicaceae, Apiaceae	Antioxidant (enzyme enhancement) Anti‐inflammatory (cytokine inhibition) Anticancer (colorectal adenocarcinoma) Hepatoprotective	Atif et al. (2025), Boulebd (2019)
**12**	Cinnamaldehyde	Cinnamon ( *Cinnamomum cassia* ), Camphor tree ( *Cinnamomum camphora* )	Lauraceae	Antimicrobial and anti‐biofilm Neuroprotective (Alzheimer's disease) Cardioprotective Antidiabetic Anti‐inflammatory	Ibi and Kyuka (2022), Luan et al. (2022)
**13**	Methyl chavicol (Estragole)	Basil ( *Ocimum basilicum* ), Tarragon ( *Artemisia dracunculus* ), Star anise ( *Illicium verum* )	Lamiaceae, Asteraceae, Schisandraceae	Antimicrobial (broad spectrum) Antioxidant (lipid peroxidation inhibition) Neuroprotective Anticonvulsant properties	Stanojevic et al. (2017), Abd Rashed et al. (2021)
**14**	Benzyl alcohol	Jasmine ( *Jasminum sambac* ), Ylang‐ylang ( *Cananga odorata* ), Tuberose ( *Polianthes tuberosa* )	Oleaceae, Annonaceae, Asparagaceae	Antimicrobial preservative Local anesthetic Anti‐anxiety properties Cosmetic applications	Stroppel et al. (2023)
**15**	Chavicol	Sweet basil ( *Ocimum basilicum* ), Holy basil ( *Ocimum sanctum* ), Clove ( *Syzygium aromaticum* )	Lamiaceae, Myrtaceae	Gastrointestinal disorders relief Antimicrobial Aromatic and culinary applications Anti‐inflammatory	Maqbool et al. (2024), Pawar et al. (2021)
**16**	Benzaldehyde	Bitter almond ( *Prunus dulcis* ), Apricots ( *Prunus armeniaca* ), Peaches ( *Prunus persica* )	Rosaceae	Anti‐arthritic Antimicrobial Flavoring agent Treatment of rheumatoid arthritis and dysentery	Durgam et al. (2023), Nazar et al. (2024)
**17**	Diosphenol	Buchu ( *Agathosma betulina* )	Rutaceae	Antiseptic (urinary tract infections) Spasmolytic Local wound antiseptic Anti‐inflammatory	Moolla and Viljoen (2008), Skosana et al. (2014)
**18**	Styrene	Liquidamber ( *Liquidambar styraciflua* )	Altingiaceae	Industrial applications Polymer production Limited biological activity reported Building construction materials	Abdel Gawad (2020), Ghavidel Darestani et al. (2018)
**19**	Cinnamyl alcohol	Camphor tree ( *Cinnamomum camphora* ), Tolu balsam ( *Myroxylon balsamum* )	Lauraceae, Papilionaceae	Perfumery and deodorant Respiratory ailments (cough preparations) Antimicrobial Flavoring agent	Monteiro et al. (2021)
**20**	Benzyl salicylate	Ylang‐ylang ( *Cananga odorata* ), Grenadine ( *Dianthus caryophyllus* )	Annonaceae, Caryophyllaceae	Aromatherapy applications UV protection Anti‐inflammatory Cosmetic fragrance (Chávez‐González et al. 2016; Gavaric et al. 2015; Papadatou et al. 2015; Sarfaraz et al. 2023)	Ozaki et al. (2015)

## 6 Commercial Products and Clinical Applications of ACEOs

Due to the known medicinal importance of ACEO, the compounds have been formulated into a large number of pharmaceutical dosage forms. In that context, topical and systemic dosage forms have been used to formulate and implicate the aromatic compounds from essential oil (ACEO) to human applications. These dosage forms are used mostly as OTC (over the counter), non‐prescription drugs, nutraceuticals, and food supplements for the management of several human disorders and for combating certain diseases. Table 3 shows the ACEOs, their dosage forms, and the applications.

**TABLE 3:** Commercial products and clinical applications of ACEO.

ACEO	Dosage forms	Application	Regulatory and traditional use summary	Composition	References
Methyl salicylate	Cream Patches Liniment	Topical analgesic for the management of all pains related to the muscles and joints	FDA's EAFUS (GRAS) FEMA No. 2467 listed GRAS Traditional dental analgesic in Ayurveda, TCM	Methyl Salicylate alone or in combination with menthol and camphor	Guo et al. (2022), Office of Dietary Supplement Programs (2009)
Diosphenol	Gel Capsules contain Buchu extract	Local antiseptic for skin wounds. Systemic antiseptic for urinary tract infection		Buchu leaves extract contains flavonoids pull diosphenol as active ingredients	Brendler and Abdel‐Tawab (2022), Moolla and Viljoen (2008), Simpson (1998)
Thymol	Gargles Mouthwas Oil Cream	Antigingivitis Disinfectant Antibacterial Antifungal products. Previously used as a vermifuge		Thyme extract contains mainly thymol or using thymol as crystals	Marchese et al. (2016), Masocatto et al. (2021), Ramadhan and Rashid (2023), Sandwith (1897)
Benzyl alcohol	Shampoos Lotions Creams Makeup	Preservative		Parenteral formulations, benzyl alcohol or with mixture of methylparaben and propylparaben is typically used.	Meyer et al. (2007)
Safrole	Oil Liniment	Parasitic infections Counterirritant		Using Sassafras, nutmeg, and camphor oils with safrole as their main ingredient	Bogusz and Al‐Tufail (2008), Clarke (2009)
Chamazulene	Tea bags Ointment	GI antispasmodics Anxiety in aromatherapy and in burns		‐ Chamomile infusion tea bags extract rich with flavonoids, bisabolol, and chamazulene. ‐ Chamomile oil or ointments are used topically	El Mihyaoui et al. (2022), Hameed et al. (2018), Rafii et al. (2020)
Guaiazulene	Sunscreen (Such as the Mid‐Day blue sun lotion) Local Pomade	Relieves acne's red, swollen phases to encourage clearer skin. Treats Recalcitrant Diaper Dermatitis		Guaiazulene as chamomile oil or as ingredient loaded in topical herbal pomade.	Akram et al. (2023), Gunes et al. (2013)
Anethole	Soap Syrup Oil Perfume Spray	Cosmetics Perfumery Food industry and Pharmaceuticals. Insect repellent	FDA GRAS FEMA GRAS	As crystals or in anise and fennel oils	Alkan and Ertürk (2020), Aprotosoaie et al. (2016), Kinghorn et al. (2010), Office of Dietary Supplement Programs (2009)
Vanillin	Powder Extract Air fresheners Floor polishes	Preservative Flavoring Sweeting agent in food and pharmaceutical industries.	FDA GRAS: GRN 1230 FEMA GRAS	As crystals, powder, or in Vanilla pod extracts. Sometimes, it is mixed with combination with other fragrances	Luu et al. (2022), Walton et al. (2003)
Eugenol	Toothpaste Powder Oil Spray	Dental market Food market (FDA GRAS, FEMA No. 2467)		It is used in clove extract, oil, or mixed with zinc oxide to create an amorphous chelate compound applied topically during endodontic therapy, cover the pulp indirectly, and temporarily fill cavities.	Markowitz et al. (1992), Ulanowska and Olas (2021)
Para cymene	Syrup Oil	Flavoring agent in food and cough syrup market		In combination with other aromatic components in oil of many odiferous plants	Elhassan (2017), Office of Dietary Supplement Programs (2009), Sahoo et al. (2020)
Myristicin	Spice	Food industry		In nutmeg and mace powders	Seneme et al. (2021)
Carvacrol	Perfume Oil	Additive Flavoring and preservatives in the food industry. Fragrance in cosmetic products.		Essential oils or aromatic plants extract	de Oliveira, Junior, et al. (2015), Javed et al. (2021), Yildirim et al. (2024)
Styrene	Polymers Polyesters Styrene‐alkyd coatings Synthetic rubbers Latex paints and coatings	In building construction fields and pharmaceutical industries		After distillation of storax, a natural balsam	IARC (1997), Miller et al. (1994)
Cinnamyl alcohol	Deodorant Perfume Sunscreen	Cosmetics industries		Cinnamon oil with other constituents as cinnamic acid and cinnamaldehyde	Letizia et al. (2005), Mahajan (2022)
Chavicol	Perfumes Flavoring Oil	Food Cosmetics industries		Included in several essential oils after their distillation in combination with other aromatics	Vargas Jentzsch et al. (2015)
Methyl chavicol	Perfumes Flavoring Oil	Food Cosmetics industries		Included in oil	Mazza and Kiehn (1992)
Cinnamaldehyde	Oil Spray Spice Flavoring extract	Odorant Flavoring Preservative in foods industries (such as candies and cookies). Repels adult mosquitoes and eliminate mosquito larvae.	GRAS spice (EAFUS spices list)	As a single ingredient liquid or in cinnamon oil with other constituents as cinnamic alcohol and cinnamaldehyde	Doyle and Stephens (2019), Siddiqua et al. (2015)
Benzaldehyde	Flavoring Gel Creams Soaps Beverage Perfume Spray	An artificial flavoring (almond and cherry) Dyes Scents (perfumes, deodorants, etc.) Pharmaceuticals Personal care goods (shave gels, moisturizing gels/creams, bath soaps, etc.) Additive for one or more tobacco product kinds.		As an individual or in some essential oils like *Prunus persica* (L.) or their extract	Cometto‐Muñiz (1981), Loch et al. (2016), Singh and Sudha (2024), Verma et al. (2017)
Benzyl salicylate	Skincare Shampoos Fragrances Toilet soaps	Cosmetics industries		Topical preparation with methyl salicylates in aromatic oils containing plants as cinnamon oil	Jantan et al. (2004), Lapczynski et al. (2007)

Abbreviations: GRAS (Generally Recognized as Safe) managed by the FDA; FEMA, Flavor and Extract Manufacturers Association.

## 7 Conclusion and Prospects

A purview of the aromatic structure‐based essential oils (ACEOs) highlighted their importance and covered the diverse set of applications in drugs, food, flavor, and pharmaceutical sectors. The overview discussed the plant‐based origins and biosynthesis of ACEOs, with a focus on numerous biotic and abiotic factors that influenced the induced production and composition of the aromatic compounds in different essential oils sourced from various plant species from different regions of the world. A comprehension of the biogenetic procedures that is essential towards maximizing the aromatic ingredients' production, designing of the biosynthetic pathways to maximize yields and quality of oil, in concordance with the aromatic constituents from different oils, and its major products have been covered. This overview also serves the purpose of an information pool of the clinical and other viable applications of the ACEOs and their ingredients in various segments, including food and flavor, cosmetics, aroma, drugs, and pharmaceutical fields. Several biological activities of the ACEOs along with their biochemical mechanisms of action have been observed, emphasized, and demonstrated. The qualities of antibacterial, antioxidant, and anti‐inflammatory nature of the essential oils and their respective aromatic constituents are presented together with the ACEOs commercial products list for their medicinal, clinical, and economic worth.

The prospects of these ACEOs lie in an intriguing option for creating novel oil and aromatic entity blends, newer oil‐based formulations as medicines, and therapeutic agents with improved and maneuverable products to the desire with design options of blends and mixing. The strength of the ACEOs in fighting infections and managing inflammation has provided them predominance over other natural ingredients and herbs in stronger applicability and appeal. The qualities of the ACEOs in food preservation have the potential to offer newer vistas in the development of better oils and aromatic constituent‐based formulations for the food industry that can be utilized in food preservation and food upkeep in diverse environments as being competent for long hours of transport, storage, and freshness of the product. The immense possibility includes the use of the ACEOs in space travel requirements for food preservation and food‐contained liquid‐gel capsules for human use. It also may usher in the development of new medicinal agents and new formulation ingredient(s) for the existing and developing drugs.

However, the challenges associated with ACEO stability, bioactivity, and the reactivity of its constituents must be addressed using nanoformulation techniques to avoid oil and constituent degradation, photoreactivity, and environmental degradation. Ensuring the safety and optimal delivery of ACEO in fixed‐dose regimens, which would enhance the applications of these products as oil and aromatic constituents, also presents challenges. Numerous researchers have attempted to address these challenges by developing dose‐calculated nanoemulsions and lipophilic nanoentities of lipid‐based encapsulates in various formats. However, standardizing drug module development and delivery requires more sophisticated efforts. Pharmacological and constituent standardizations of ACEOs are required, particularly for aromatic constituents that lack pharmacological standardization, to confirm the single or multiple activities of various oils from different sources. To validate the activity and synergy levels of the bioactivity being tested for various oils and their aromatic constituents, the primary aromatic constituents should be standardized both individually and in combination with other constituents in varying proportions.

Thus, the ACEOs are highly valued, economically and medicinally important, also owing to their diversity of aromatic ingredients they provide, which extends beyond their sensory qualities to include their functional advantages as chemical entities in diverse applications in foods, flavor, fragrance, cosmetics, and therapeutics sectors. The market for materials containing ACEOs is projected to rise exponentially, as consumers look for safe and efficient substitutes, and industry demands more and better alternatives in the essential oils domain for its varied applications of upgraded and robust quality of aromatic essential oils and their linked constituents.

## Author Contributions

**Hamdoon A. Mohammed:** conceptualization (equal), writing – original draft (equal), writing – review and editing (equal). **Ghassan M. Sulaiman:** conceptualization (equal), writing – original draft (equal), writing – review and editing (equal). **Ali Z. Al‐Saffar:** software (equal), writing – original draft (equal), writing – review and editing (equal). **Mayyadah H. Mohsin:** software (equal), writing – review and editing (equal). **Riaz A. Khan:** conceptualization (equal), writing – original draft (equal), writing – review and editing (equal). **Noora A. Hadi:** writing – original draft (equal), writing – review and editing (equal). **Shahad Basil Ismael:** writing – original draft (equal), writing – review and editing (equal). **Fatma Elshibani:** writing – original draft (equal), writing – review and editing (equal). **Ahmed Ismail:** writing – original draft (equal), writing – review and editing (equal). **Mosleh M. Abomughaid:** writing – original draft (equal), writing – review and editing (equal).

## Conflicts of Interest

The authors declare no conflicts of interest.

## Data Availability

The authors have nothing to report.
